# Protein secondary structure assignment revisited: a detailed analysis of different assignment methods

**DOI:** 10.1186/1472-6807-5-17

**Published:** 2005-09-15

**Authors:** Juliette Martin, Guillaume Letellier, Antoine Marin, Jean-François Taly, Alexandre G de Brevern, Jean-François Gibrat

**Affiliations:** 1 INRA, Unité Mathématiques Informatique et Génome, Domaine de Vilvert, 78352 Jouy en Josas Cedex, France; 2 INSERM U726, Equipe de Bioinformatique Génomique et Moléculaire, Université Paris 7, case 7113, 2 place Jussieu, 75251 Paris cedex 05, France

## Abstract

**Background:**

A number of methods are now available to perform automatic assignment of periodic secondary structures from atomic coordinates, based on different characteristics of the secondary structures. In general these methods exhibit a broad consensus as to the location of most helix and strand core segments in protein structures. However the termini of the segments are often ill-defined and it is difficult to decide unambiguously which residues at the edge of the segments have to be included. In addition, there is a "twilight zone" where secondary structure segments depart significantly from the idealized models of Pauling and Corey. For these segments, one has to decide whether the observed structural variations are merely distorsions or whether they constitute a break in the secondary structure.

**Methods:**

To address these problems, we have developed a method for secondary structure assignment, called KAKSI. Assignments made by KAKSI are compared with assignments given by DSSP, STRIDE, XTLSSTR, PSEA and SECSTR, as well as secondary structures found in PDB files, on 4 datasets (X-ray structures with different resolution range, NMR structures).

**Results:**

A detailed comparison of KAKSI assignments with those of STRIDE and PSEA reveals that KAKSI assigns slightly longer helices and strands than STRIDE in case of one-to-one correspondence between the segments. However, KAKSI tends also to favor the assignment of several short helices when STRIDE and PSEA assign longer, kinked, helices. Helices assigned by KAKSI have geometrical characteristics close to those described in the PDB. They are more linear than helices assigned by other methods. The same tendency to split long segments is observed for strands, although less systematically. We present a number of cases of secondary structure assignments that illustrate this behavior.

**Conclusion:**

Our method provides valuable assignments which favor the regularity of secondary structure segments.

## Background

In 1951, Pauling and Corey predicted the existence of two periodic motifs in protein structures: the *α*-helix [1] and the *β*-sheet [2] which turned out to be major features of protein architecture. Secondary structures, because they allow a simple and intuitive description of 3D structures, are widely employed in a number of structural biology applications. For instance, they are used for structure comparison [3] and structure classification [4,5]. They also provide a natural frame for structure visualization [6,7].

In recent years, secondary structures have come to play a major role in a number of methods aiming at predicting protein 3D-structures. Indeed, being able to predict accurately secondary structure elements along the sequence provides a good starting point toward elucidating the 3D-structure [8,9]. Current algorithms for predicting the secondary structure provides accuracy rates of about 80% for a 3 state prediction: *α*-helix, *β*-strand and coils [10-12], using neural networks and evolutionary information. The maximum achievable prediction has been estimated to lie in the range 85% [13] to 88% [14].

The divergence between observed and predicted secondary structure has been noticed early [15]. It took more time, though, for the structuralist community, to realize that obtaining an accurate and objective secondary structure assignment was not a trivial task, due to the variations observed in secondary structures when compared to ideal ones. As noted by Robson and Garnier [16]: "In looking at a model of a protein, it is often easy to recognize helix and to a lesser extent sheet strands, but it is not easy to say whether the residues at the ends of these features be included in them or not. In addition there are many distorsions within such structures, so that it is difficult to assess whether this represents merely a distortion, or a break in the structure. In fact the problem is essentially that helices and sheets in globular proteins lack the regularity and clear definition found in the Pauling and Corey models." For instance, as found by Barlow and Thornton [17] and Kumar and Bansal [18,19], a majority of *α*-helices in globular proteins are smoothly curved. Therefore, a group of experts (NMR spectroscopists and crystallographers), asked to assign the secondary structure of a particular protein, is likely to come up with different assignments.

To cope with this problem, as well as the increase in the number of experimentally solved 3D structures, the need for automatic secondary structure assignment programs was felt in the mid seventies. Such programs are intended to embody expert's knowledge and to provide consistent and reproducible secondary structure assignments. Periodic secondary structures generate regularities that can be used as criteria to define them, e.g., C*α *distances, dihedral angles, like *α *angles or pairs of (Φ/Ψ) angles, and specific patterns of hydrogen bonds. Along the years, various methods using these criteria have been proposed. The first implementation of such methods, allowing automatic secondary structure assignment from 3D coordinates, was done by Levitt and Greer [20]. The algorithm was mainly based on inter-C*α*; torsion angles.

A few years later, Kabsch and Sander developed a method called DSSP [21] that still remains one of the most widely-used program for secondary structure assignment. The DSSP algorithm is based on the detection of hydrogen-bonds defined by an electrostatic criterion. Secondary structure elements are then assigned according to characteristic hydrogen-bond patterns. This methodology has been widely accepted as the gold standard for secondary structure assignment. A number of software packages make use of DSSP when they need to assign secondary structures. For instance rasmol [6], the most widely distributed visualization software, assigns the repetitive structures with a fast DSSP-like algorithm. Similarly GROMACS analysis tools use the DSSP software [22].

STRIDE [23] is a software related to DSSP. It makes a very similar use of hydrogen-bond patterns to what is done in DSSP, although the definition of hydrogen-bonds is slightly different. In addition STRIDE takes into account (Φ/Ψ) angles to assign secondary structures. STRIDE is used by the visualization tool VMD [7] to assign secondary structures.

SECSTR [24] belongs to the same family of methods. It has been developed specifically to improve the detection of *π*-helices. Indeed, SECSTR's authors found dssp and STRIDE unable to detect several *π*-helices they were able to characterize with their method.

Other methods have been developed that use different criteria to assign secondary structures. DEFINE [25] relies on C*α *coordinates only and compares C*α *distances with distances in idealized secondary structure segments. It also provides a description of super-secondary structures. P-CURVE approach [26] is based on the definition of helicoidal parameters for peptide units and generates a global peptide axis. PSEA [27] only considers C*α *atoms. It is based on distance and angle criteria. XTLSSTR [28] has been developed to assign secondary structures " in the same way a person assigns structure visually", from distances and angles calculated from the backbone geometry. It is concerned with amide-amide interactions. The most recent method, to the best of our knowledge, is VoTAP [29] which employs the concept of Voronoi tessellation, yielding new contact matrices.

Let us notice that structure files provided by the Protein Data Bank (PDB) [30] contain secondary structure descriptions in the HELIX, SHEET and TURN fields (see the PDB Format Description Version 2.2 [31]). These secondary structure descriptions are either provided by the depositor (optional) or generated by DSSP. Approximately 90% of the PDB files do have secondary structure fields. However, even though these fields are used, it may happen that only a few secondary structure elements, of interest for the depositor, are described, the others being ignored.

The variety of available methods illustrates the fact that there are several legitimate ways to define secondary structures. It is hardly surprising that these different methods provide different assignments, especially at the edges of secondary structure segments. For example, Colloc'h and co-workers [32] showed that the percentage of agreement is only 63% between DSSP, P-CURVE and DEFINE and that DEFINE tends to assign too many repetitive secondary structure segments. XTLSSTR authors noted that DSSP assigns more *β*-strands than XTLSSTR does [28]. SECSTR is logically more sensitive for *π*-helix detection than DSSP or stride [24].

In this paper we want to focus on how well some of the above methods handle the secondary structure irregularities mentioned by Robson and Gamier [16]. We are particularly interested in the way these different methods process the edges of secondary structure elements and deal with the various structure distorsions occurring in proteins. For structures solved by X-ray diffraction, it is well known that the resolution has a direct effect upon the quality of the resulting model. One expects the secondary structure assignment to be less accurate for low resolution structures [23]. It is thus interesting to assess the effect of the resolution upon the secondary structure assignment proposed by the different methods. It is also worth comparing secondary structure assignments for structures solved by X-ray crystallography and by NMR techniques. Structures solved by NMR correspond to proteins in solution and provide a more "dynamic" representation of the protein conformation than X-ray structures do. NMR structures are therefore more prone to local distorsions and constitute difficult, and interesting, cases for secondary structure assignment methods.

In the following we present a new method for secondary structure assignment, called KAKSI (KAKSI means "two" in Finnish) based on C*α *distances and (Φ/Ψ) angles. These characteristics are intuitively used when examining visually a 3D structure. Our main purpose in developing this method was to deal, in a satisfactory way, with the structure irregularities. For instance we consider that regions of the polypeptide chain that show an abrupt change in their curvatures (such as kinks in *a *helices) should be considered as breaks in periodic secondary structures. The objective of an assignment method is to provide accurate and reliable assignment. Demonstrating that our methodology is an improvement over existing methods would be difficult since there is no standard of truth to benchmark methods with. We then carry out comparisons of the assignments of this new method with a number of other methods that use different criteria to define secondary structures: DSSP, STRIDE, SECSTR, XTLSSTR and PSEA, as well as with the descriptions found in PDB files. These comparisons are performed on 4 different datasets: 3 X-ray datasets with, respectively, high, medium and low resolution and an NMR dataset. This allows us to evaluate the effect of the resolution and experimental method upon the different secondary structure assignment methods.

We address the problem of inclusion of residues at the edges of helices and strands by examining the length of segments assigned by different methods. We also study the problem of correctly defining segments in case of distortions. More specifically, for helices, we appraise the geometry of helical segments using HELANAL [33], a software dedicated to this task.

Finally, we illustrate how KAKSI deals with distorted secondary structures by comparing its assignments with STRIDE assignments for a number of difficult cases.

## Results and discussion

### KAKSI parameters

In KAKSI secondary structure detection depends on a number of parameters (see Method section).

To test the robustness of the method to the choice of these parameters, we examined the effect of changing *ε*_*H*_, *ε*_*b *_and *σ*_*b *_upon the secondary structure contents of the *comparison sets*. We let *ε*_*H *_and *ε*_*b *_vary in the range 1.29 to 3.30, and *σ*_*b *_in the range 3 to 6. Each parameter is tested separately, while keeping other parameters to the selected values given in Methods section.

The effects are similar on all sets of structures. The decrease of *ε*_*H *_below 1.96 results in a moderate diminution of the percentage of *α*-helix, whereas this percentage slightly increases when *ε*_*H *_is greater than 1.96. Fewer *β*-sheets are assigned when *ε*_*b*_, is lower than 2.58. On the contrary, the percentage of *β*-sheets increases when *ε*_*b*_, is greater than 2.58. Slightly more *β*-sheets are assigned when *σ*_*b *_is lower than 5, and there is a diminution of *β*-sheets assignment when *σ*_*b *_is greater than 5.

Two different behaviors are observed: KAKSI assignments are not very sensitive to variations of *α*-helix detection thresholds, but quite sensitive to variations of *β*-sheets detection thresholds. This is easily explained by the detection heuristic: the detection of *α*-helix is achieved by the distance or the angle criteria, moderate changes of *ε*_*H *_are balanced by other criteria. On the contrary, the *β*-sheet detection is achieved by the satisfaction of both, distance and angle, criteria.

The two criteria implemented in KAKSI for kink detection in *α*-helices, K1 based on (Φ/Ψ) angles and K2 based on axes, are also tested. To evaluate the efficiency of each criterion, we analyze the geometry of kinked helices with the HELANAL software. We monitor the fraction of helices classified as kinked by HELANAL. This fraction is reduced when each criterion is used separately showing that both criteria are able to detect kinks (data not shown). Results obtained with K1 agree better with HELANAL results than those obtained with K2. However the best agreement with HELANAL is obtained when criterion K1 and K2 are used sequentially. Hereafter, KAKSI assignments are obtained with the parameter values given in Material and Methods and both criteria K1 and K2 applied for kink detection.

### Secondary structure content

The secondary structure content is used to assess the sensitivity of different assignment methods to the structure resolution. Table 2 shows the secondary structure content in all our *comparison sets*, according to five available assignment softwares, KAKSI and the PDB description.

There is no absolute consensus, even for the *HRes set*, about secondary structure content according to different methods. STRIDE and DSSP figures are very close, as expected due to the similarity of these methods [21,23]. PSEA systematically assigns less helices and more strands than other methods. PDB assignments are always richer in *α*-helix than any automatic procedure. KAKSI assigns a fraction of periodic secondary structures comparable to STRIDE and DSSP on the *HRes set*.

Secondary structure contents in the *HRes *and the *MRes sets *are similar according to different methods. Assignments on the *LRes *and the *NMR sets *result in smaller contents in regular secondary structures. This is true for every assignment methods, but more or less marked, depending on the method. *β*-assignment is lower on the *LRes set *for a majority of methods. Only PSEA assignments show a proportion of *β *comparable for all datasets. It must be noted that this method consistently assigns more *β*-strands than all other methods, whatever the dataset considered. Overall, though, the influence of the resolution upon the assignments of the methods is moderate. The type of technique use to solve the structure (X-ray vs NMR) appear to have a more pronounced effect.

The decrease in *β*-sheets assignment on the *LRes *and *NMR sets *indicates that less stringent parameter values are required when dealing with structures belonging to these sets. For example, KAKSI assignment on the *LRes set *with *σ*_*b *_= 3 result in a proportion of 22.3% residues in *β*-sheet and 20.7% with *σ*_*b *_= 3.30 (data not shown). In the same way, the percentage of *β*-sheet residues in the *NMR sets *is about 17.7% with *σ*_*b *_= 3 or *ε*_*b *_= 3.30. Consequently, we suggest to adapt the *β*-sheet detection parameters when dealing with low resolution and NMR structures.

### Measures of global agreement between methods

#### *C*_3 _scores

Table 3 shows the *C*_3 _scores obtained for the *HRes set *(the overall agreement between the different assignment methods show the same tendencies for the different *comparison sets*, [see Additional file 1]). A group of methods shows a strong agreement: *C*_3 _scores within the group DSSP, STRIDE, SECSTR and PDB are all in the range 87.4% (SECSTR versus PDB) to 95.4% (STRIDE versus DSSP). The strong similarity between DSSP and STRIDE assignments, which both used a hydrogen-bond criterion, has been noted in previous studies [27,29,34]. The SECSTR method is strongly related to the DSSP algorithm and logically belongs to this group. As was expected, PDB descriptions are very close to DSSP assignments due to the way secondary structure assignments are performed.

Assignments given by XTLSSTR are the most different from others: *C*_3 _scores with DSSP, STRIDE, SECSTR and PDB are all below 81%. KAKSI and PSEA show an intermediate behavior of the other methods [see Additional file 2]. The *C*_3 _scores are all in the same range, between 81.5% (KAKSI/PSEA) and 83.5% (KAKSI/STRIDE), excluding XTLSSTR (78.3%).

#### SOV criterion

The SOV criterion is usually employed for secondary structure prediction evaluation, whereas here, comparisons are made between alternative structure assignments. SOV values depend on which structure ischosen as reference. To allow comparison, KAKSI is taken as reference. Table 4 shows SOV values computed from the *HRes set *for helices and strands, between KAKSI and other methods. SOV values for other datasets are available, [see Additional file 3].

For helical segments, the highest SOV with KAKSI assignment is obtained with DSSP (91.7%). It lies in the same range for STRIDE. It is slightly lower for other methods but remains above 87%. For the strands, a good agreement is seen with DSSP, STRIDE and PDB (SOV scores about 90%). Lower SOV (about 83%) are found with PSEA and SECSTRC. Moderate agreement is seen with XTLSSTR (75.8% only). *C*_3 _score between XTLSSTR and KAKSI is only 78.3%(see table 3). SOV values are high for helices and slightly lower for strands, showing that differences between both methods mainly concern *β*-sheets assignments. Hereafter we will restrict our comparisons to KAKSI, STRIDE, and PSEA assignments on the *HRes set*. STRIDE is a widely-used method whose results are very similar to DSSP and PDB, as shown by the *C*_3 _scores. STRIDE is chosen because it exhibits the largest *C*_3 _score with KAKSI. PSEA is chosen because its algorithm fairly differs from other methods, but SOV values remain consistent when compared to KAKSI'S.

#### Segment length distribution

The length distributions of helices and strands assigned by KAKSI, PSEA and STRIDE on the *HRes set *are shown on Figure 3.

In helix distributions, three zones can be distinguished. (i) For helices shorter than 8 residues, the distributions are very different: STRIDE assigns many 3 residue long helices, whereas PSEA and KAKSI do not assign helices shorter than 5 residues. PSEA assignments results in slightly larger number of short helices than STRIDE. KAKSI distribution shows a very high peak at 7 residues. (ii) In the range 8 to 15 residues, small differences are observed: KAKSI distribution shows a peak about 12 residues, unlike PSEA and STRIDE distributions. (iii) For helices longer than 15 residues, distributions are similar.

Similarly, 3 distinct zones appear in the strand distributions. (i) Up to 6 residues, PSEA and KAKSI curves show larger peaks than STRIDE distribution, at 3 to 5 residues for KAKSI, and 4 and 5 residues for PSEA. PSEA and KAKSI do not assign strands shorter than three residues, whereas STRIDE assignment result in a large number of 1-residue long strands. These segments are isolated *β*-bridges (state b in stride assignments). (ii) Between 6 and 9 residues, psea and KAKSI segments are more numerous than STRIDE segments. (iii) After 9 residues, the distributions are identical.

Global measures, such as *C*_3 _and SOV scores, show that KAKSI assignments are globally consistent with those given by other existing methods. The length distributions of helices and strands indicates that segment distribution is also roughly similar across methods. This broad consensus was expected. In the following sections we now turn toward the study of details of the assignments, in particular, as mentioned in the introduction, we compare the way different methods deal with the edges of secondary structures and cope with local distorsions.

### Detailed comparison

#### Pair length

The SOV criterion is a measure of the global overlapping of secondary structure segments. It gives no information about the effect of length of segments or about the respective length of facing segments. Figure 4 shows the plot of lengths for pair of corresponding repetitive structure segments between STRIDE and KAKSI, and PSEA and KAKSI assignments. The pairs are those used for the SOV computation: a pair is considered when there is at least one residue in the same state for the two assignments. Unpaired segments are ignored.

Taking KAKSI assignment as our reference, three different cases occur: (i) One segment according to KAKSI corresponds to a single segment in another method assignment: these are *one-to-one events*. (ii) One segment assigned by KAKSI corresponds to two or more segments in another method assignment. We call this a *fusion event*. (iii) The symmetric case, several segments in KAKSI assignment corresponding to a single segments in another method assignment, is called a *division event*. The three cases are available plotted on separate graphs [see Additional file 4].

#### Helix length

The strong accumulation of points along the diagonal, on both plots (KAKSI versus STRIDE and KAKSI versus PSEA) and for every segment lengths shows that KAKSI often agrees with other methods about the length of helices. There are more points below the diagonal than above, indicating that KAKSI tends to assign slightly longer segments than STRIDE and PSEA (one or two residue longer). This occurs for all segment lengths, but it is more striking on the PSEA/KAKSI comparison.

The points appearing far from the diagonal correspond to *division *and *fusion events*, as shown by the squared correlation coefficients *r*^2^. Correlations are calculated on the pairs (PSEA or STRIDE length/KAKSI length) and are used as indicators for the dispersion about the diagonal. On the KAKSI/STRIDE comparison, *r*^2 ^= 0.28 for all the 5146 pairs, but reaches 0.88 when only the 3755 *one-to-one events *are considered. The remaining pairs correspond to 142 cases of *fusion *and 1249 cases of *division events. Division events *are responsible for the numerous observations of pairs of short helices in KAKSI assignment (5 to 9 residues) with longer helices in PSEA and STRIDE assignments (10 to 20 residues).

Similarly, for the KAKSI/PSEA comparison there are 4762 pairs (*r*^2 ^= 0.23), distributed in 3443 *one-to-one events *(*r*^2 ^= 0.85), 150 *fusion *and 1169 *division events*. Numerous cases of divisions appear on the plot as pairs of 5 to 9 residue helices for KAKSI and 10 to 20 residue helices for PSEA.

For both comparisons (KAKSI/STRIDE and PSEA/KAKSI), the number of *division events *is greater than the number of *fusion events*, showing that KAKSI tends to split long segments into shorter ones. This is a direct consequence of the kink detection mechanism used in KAKSI. It also explains why short helices are more abundant in KAKSI assignments than in STRIDE and PSEA. Some examples of this phenomenon are illustrated in Fig 5.

#### Strand length

The situation is less clear than for helices. The points are more dispersed and there is no clear accumulation of points accounting for *division events*. In the KAKSI/STRIDE comparison, the 5974 pairs yield a *r*^2 ^equal to 0.35. This value increases to 0.69 when only the 5403 *one-to-one events *are considered. Amongst the remaining pairs 214 correspond to *fusion events*, and 357 to *division events*. The splitting of long segments is thus less systematic than for helices. This makes senses since there is no mechanism similar to the kink detection in helices for *β*-strands. 52% of the *one-to-one events *fall above the diagonal (longer segments in KAKSI assignment) and 22 % fall below the diagonal (shorter segments in KAKSI assignment). The remaining 26% are on the diagonal. It shows that KAKSI tend to assign longer strands than STRIDE.

In the KAKSI/PSEA comparison, *r*^2 ^equals 0.23 on the 5041 pairs and 0.44 on the 4694 *one-to-one events*. There are 214 *fusion events *and 133 *division events*. The numbers of *division *and *fusion *events are close, indicating that there only a slight splitting effect. 27% of the *one-to-one events *are on the diagonal, 50% are above (greater length in PSEA assignment) and 23% are below (greater lenght in *kaksi *assignment). In a majority of case, KAKSI assigns shorter strand segments concerning *one-to-one events*.

For both kind of segments and both comparisons, we also checked for the existence of systematic shifts of the segments toward the N-ter or C-ter termini of the secondary structure elements. No such systematic bias was found (data not shown).

#### Helix geometry analysis with HELANAL

In KAKSI we pay a special attention to the detection of kinks in *α*-helices by applying angle and axis criteria. This motivates the study of the geometry of helices with an external tool, according to alternative definitions of helix locations. We check the geometry of helices assigned by the different assignment methods with the HELANAL software. We are interested in the distribution of helices into the three classes: linear (L), curved (C) or kinked (K). Unclassified helices represent less than 1% in our datasets.

When analyzed by HELANAL, helices assigned by all methods show a high proportions of kinks. On the *HRes set*, for example, about 20% (DSSP, STRIDE, KAKSI) up to 30% (SECSTR, XTLSSTR) helices appear classified as kinked. This ratio is 16% only for the PDB assignments, and less than 10% for PSEA. When the resolution gets worse, this proportion increases [see Additional file 5]. On the *NMR set*, we observe as much as 40% kinked helices for PSEA assignment and more 50% kinked helices for STRIDE, SECSTR and PDB.

This high ratio of irregular helices (curved or kinked) is in agreement with previously published results [17]. However, the high ratio of kinked helices found here is larger than previously reported by Kumar and Bansal [19]. There is a difference between Kumar and Bansal's work and our study: they modified helix assignment given by DSSP before submission to HELANAL. Using distance and axis criteria, they corrected helix boundaries to avoid distortions at the termini. Consequently, the high ratio of kinked helices is likely due to these terminal residues. Rather than applying the correction used by Kumar and Bansal, we apply a systematic correction before submitting helices to HELANAL, i.e., one residue is removed at each helix terminus. The reason for applying a systematic correction rather than a correction based on geometrical criteria is that we want to make a statistical comparison of helices assigned by various softwares. The goal is not to correct potentially wrong helices boundaries. We want to evaluate the assignments as they are produced by the softwares and used in later applications.

Table 5 shows the results obtained on the *HRes set*, before and after correction, for helices defined by the seven methods. Results for other datasets are available [see Additional file 5].

As HELANAL can handle only helices longer than nine residues, we restrict our analysis to helices longer than eleven residues. When removing the first and last residues of helices, the ratio of kinked helices decreases, showing that part of the kinks are due to distortion at the termini. After correction, the geometry of helices assigned by KAKSI (14.5% of kinked helices) is the closest to the geometry of helices described in the PDB (12% kinked helices). The KAKSI method also assigns the highest ratio of linear helices (12.3%). PSEA has only 7.8% kinked helices but it should be noted that the number of helices submitted to analysis is slightly lower.

It is interesting to investigate the geometry of helices when KAKSI assigns several helices in a region where STRIDE assign a single long helix, i.e., the *division events*. If we consider the *division events *involving pair of helices longer than nine residues, we find 128 pairs where a kinked helix assigned by *stride *corresponds to curved or linear helices assigned by KAKSI. The symmetric case, kinked helices in KAKSI assignment paired with a curved or linear helices in STRIDE assignment concerns only 7 cases. This indicates that splitting long helices into several short ones helps to define helices devoid of kink.

All these observations suggest that the kink detection implemented in KAKSI is efficient and leads to more reliable helix locations. The major feature of KAKSI assignments is then the geometry of *α*-helices: while assigning slightly longer helices than stride, the global geometry of helices remains satisfactory, with more linear helices than other assignments and a limited ratio of kinked helices, very close to PDB assignments. This is accomplished by dividing long distorted helices when appropriate. Some examples are shown in the following section.

### Some examples of assignment disagreements

Figure 5 shows some interesting examples of disagreement between STRIDE and KAKSI assignments. The first three examples in Figure 5 concern disagreement about helix assignments. In example (a), the long helix assigned by STRIDE shows a sharp kink. In KAKSI assignment it is replaced by two helices from residues 4 to 19 and 21 to 34. The first helix is classified as curved by HELANAL. The second one is classified as kinked, but it becomes linear after removal of terminal residues. The angle between two global axes fitted in these two helices is 83°. The second example (b), is even more striking: a 33-residue long helix defined by STRIDE from residues 308 to 340 exhibits a reverse turn near its N-terminal edge. The definition given by KAKSI is two helices from 308 to 315 and 320 to 341. The first helix is too short to be analyzed by HELANAL and the second one is classified as linear. The third example is the case of a division of a long helix assigned by STRIDE into three segments in KAKSI assignment. Although less marked than for the first two examples, the kinks are well apparent. The three helices defined by KAKSI are all classified as curved by HELANAL, with their global axes making angles equal to 135 and 120° between the first and the second, and the second and the third helix respectively.

The last example 5(d) is an example of disagreement on a *β*-strands assignment. *β*-strands assigned by STRIDE are fairly curved, allowing a change of direction of the backbone. No specific routine is implemented in KAKSI to split distorted strands, as it is done for helices. Nonetheless, the criteria of *β*-sheet assignment being fairly strict, some cases of division in long *β*-strands can also occur. These examples illustrate the fact that a small disagreement on a per-residue basis can result in a radical change in the structure description. In the examples shown on Fig. 5 we believe that KAKSI assignments provide a more pertinent description of the protein structure.

## Conclusion

We have developed a new automatic procedure to assign secondary structures from 3D coordinates. Our method, KAKSI, uses C*α *distances and (Φ/Ψ) angles and pay a special attention to kink detection in helices. Like other methods (except PSEA), it is sensitive to the resolution, and the type of experimental technique used to solve the structure. Consequently, we propose to choose detection parameters according to the structure resolution or technique and the nature of the secondary structure, since *β*-sheets are more difficult to detect. The careful comparison of KAKSI assignments with assignments produced by five available methods and the description provided by the PDB highlights the similarities and differences between the different methods. Good general agreement are observed between methods, especially on *α*-helices. The length of *α*-helices and *β*-strands, in case of agreement on the number of segments, are very similar when compared to STRIDE and PSEA. When different lengths are assigned, we observe slightly longer *α*-helices and *β*-strands than the STRIDE definition. When two methods disagree on the number of segments, we observe more *division events *than *fusions*, i.e., several short helices assigned by KAKSI in front of a unique long helix assigned by STRIDE or PSEA. *Division events *are also slightly predominant in the comparison of *β*-strand length with STRIDE and PSEA. The study of *α*-helix geometry with an external tool reveals that KAKSI helices are less kinked that helices assigned by other methods, except PSEA. KAKSI is also the method that assigns helices with geometrical characteristics in best agreement with helices described in the PDB, and, maybe more important, the highest proportion of linear helices. As stated by Andersen and co-workers [35], each method reflects its own definition of secondary structures. Our definition favors a certain regularity of secondary structure elements, as illustrated by the examples on Fig. 5.

## Methods

### Datasets

The KAKSI method uses geometrical characteristics of *α*-helices and *β*-sheets extracted from available protein structures. A reference set (*Ref set*), consisting of 2880 structural domains taken from ASTRAL 1.63 [36] is used to estimate these geometrical characteristics. The list of domains with less than 40% identity provided by the ASTRAL server [37] is filtered to keep only X-ray structures with a resolution better than 2.25 Å and longer than 50 residues.

KAKSI assignments are compared with secondary structure assignments done by other methods. For the reasons mentioned above four different sets of structures are used. Hereafter we refer to these datasets as the *Comparison sets*.

The number of structures reported below refer to the files that are successfully processed by all assignment programs and contain a secondary structure description provided by the PDB.

• A High Resolution set (*HRes set*): X-ray structures with resolution better than 1.7 Å, R-factor < 0.19, identity percentage between sequences less than 30%, obtained from the WHATHIF website [38,39]. There are 689 structures in this set, corresponding to 151922 residues with a defined secondary structure, i.e., excluding missing coordinates.

• A Medium Resolution set (*MRes set*): X-ray structures with resolution between 1.7 Å and 3 Å, R-factor < 0.3, identity percentage between sequences less than 30%, minimum length of 40 residues, provided by the PISCES website [40,41]. There are 624 structures in this set, corresponding to 160 276 residues with a defined secondary structure.

• A Low Resolution set (*LRes set*): X-ray structures with resolution worse than 3 Å, R-factor > 0.3, identity percentage between sequences less than 30%, minimum length of 40 residues, provided by the PAPIA website [42]. There are 332 structures in this set, corresponding to 97852 residues with a defined secondary structure.

• A *NMR set*: structures with less than 30% sequence identity, extracted from all NMR entries obtained on the PDB website [43]. The redundancy of the set is reduced to 30% sequence identity with PISCES. There are 296 structures in this set, corresponding to 27533 residues with a defined secondary structure.

These lists are available on the web [see Additional file 6].

### KAKSI method

The assignment of repetitive secondary structures by KAKSI is based on a set of characteristic values of C*α *distances and (Φ/Ψ) dihedral angles. The parameters of KAKSI have been chosen to best fit the secondary structure assignments obtained from the PDB files (HELIX and SHEET fields). These fields, when present, are automatically generated with the DSSP method or are provided by the depositor who might have used some secondary structure assignment program and/or might have inspected visually the 3D structure and assigned himself the secondary structures. We use these PDB assignments as our gold-standard for the sake of parameter calculations, keeping in mind that the data are partly similar to DSSP assignments. Assignment is done by sliding windows along the sequence. *α*-helices are assigned first, followed by *β*-sheets. Two windows are slid for the *β*-sheet detection because we only want to assign *β*-strands involved in *β*-sheets. Residues once assigned in *α*-helix cannot be re-assigned in *β*-sheets.

#### Secondary structure characteristics used by the KAKSI heuristic

As mentioned earlier, *α*-helices and *β*-strands being periodic structures, their backbone geometry exhibits a number of regularities. This periodicity leads to characteristic distances between C*α *atoms as well as characteristic values of (Φ/Ψ) dihedral angles.

**Figure 1:**
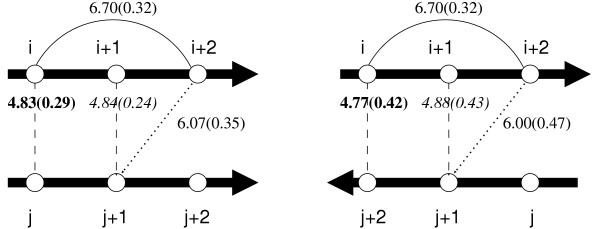
**Typical C*α *distance in *β*-sheets**. Typical C*α *distances computed from the *Ref set *in parallel (left part) and anti-parallel *β*-sheets. Mean distances are indicated in Å with their standard deviations within parentheses. Separate statistics were computed for distances involving only residues in strand cores (italic) and distances involving residues at strand edges (bold). For the intra-strand distance (type *i *to *i *+ 2), no distinction is made on the sheet orientation.

More precisely, we have estimated from the *Ref set*:

• *distances between Cα in α-helices and β-sheets*. Different statistical distributions are computed for terminal residues and cores of secondary structure segments because greater variations are observed at segment termini. For *α*-helices, 4 distances are considered between residues *i *and *j *along the sequence, with *j *∊ [*i *+ 2, *i *+ 5]. Table 1 shows the means and standard deviations obtained on the *Ref set*. For *β*-sheets, three different types of distances are considered. Figure 1 illustrates these distances and reports the values obtained on the *Ref set*.

• (Φ/Ψ) *values for residues involved in α-helices and β-strands*. Densities of (Φ/Ψ) angles are computed using Ramachandran maps. These maps are divided into 10 by 10 degree squares. This yields two *population maps*: one specific of *α*-helices and the other specific of *β*-strands [see Additional file 7]. For the *α*-helix *map*, we only consider angles lying in the area (Φ < 0° and -90° < Ψ < 60°) and we set to zero square frequencies that are too low (frequency <*δ*_*H*_). In this study, the threshold *δ*_*H *_is fixed, empirically, to 20 × *n*_*mean*_, *n*_*mean *_being the mean frequency for a square in the Ramachandran map.

As mentioned above we are particularly interested in the detection of kinks in *α*-helices. Kinks are frequent and not easy to detect with usual distance and angle criteria. In a regular helix, (Φ/Ψ) angles should remain located in a narrow region of the Ramachandran map. One way to detect kinks (criterion K1 below), is to compute distances between (Φ/Ψ) pairs of successive residues *j *and *j + *1 in the Ramachandran map. We use the 95-percentile of the distance distribution in *α*-helices. The kink detection is only performed in helix cores, terminal residues of segments being disregarded in the computation.

#### KAKSI heuristic for helix and strand assignment

**Figure 2:**
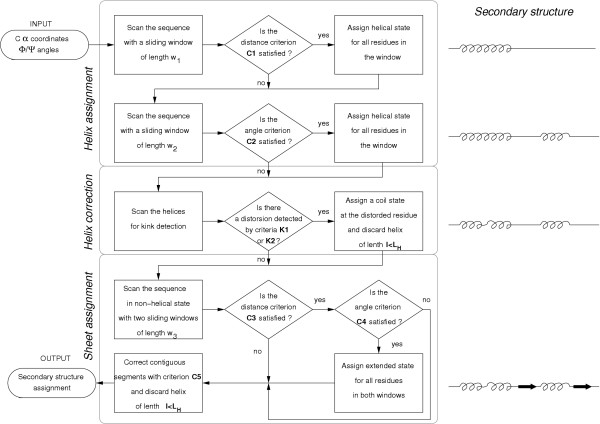
**Flow-chart of the kaksi heuristic for secondary structure assignment**. Minimum length for helices is set to *L*_*H *_= 5. The criteria C1, C2, C3, C4, C5, K1 and K2 are detailed in the text.

**Figure 3:**
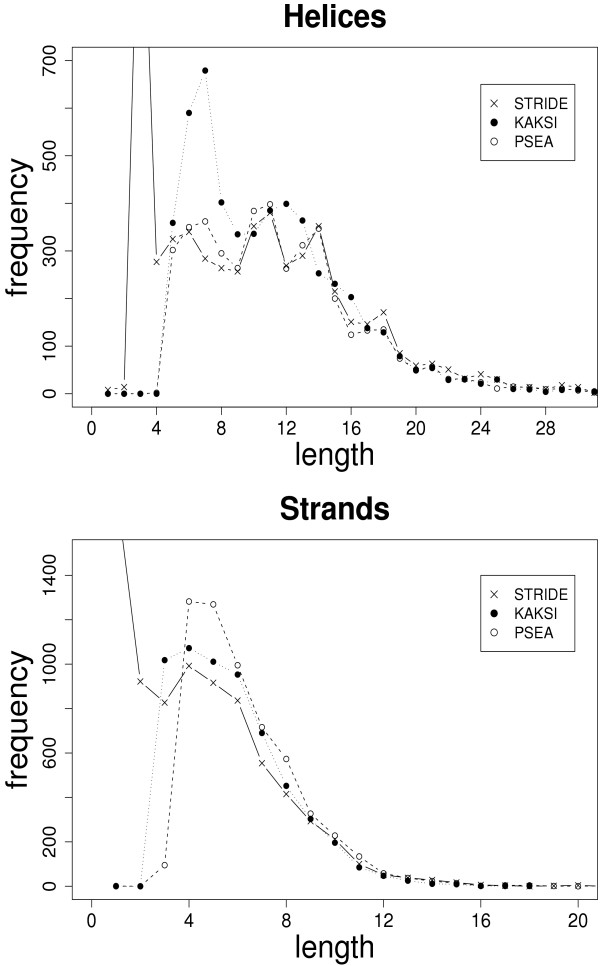
**Length distribution of helices and strands assigned by stride, psea and kaksi**. Length distribution of helical (top) and extended (bottom) segments assigned by STRIDE (plain line and crosses), PSEA (dashed line and open circles), and KAKSI (dotted line and filled circles), on the *HRes set*. The STRIDE assignment generates a large number of 3 residue-long helices (1238 segments) and 1 residue-long strands (corresponding to 1800 *β*-bridges).

**Figure 4:**
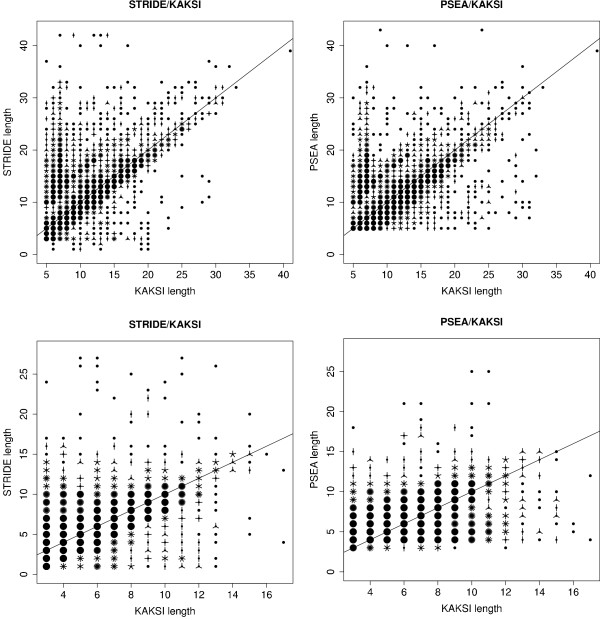
**Length for pair of segments assigned by stride vs kaksi and psea vs kaksi**. Length for pair of helices (upper part) and strands (lower part) when comparing STRIDE and KAKSI assignments, and PSEA and KAKSI assignments. We report a pair when we found at least one residue in the same state in both assignments. Data are shown as a "sunflower plot": a point stands for a single observation, then the number of "leaves" is proportional to the number of additional observations. The diagonal x = y (same length for two assignments) is shown.

**Figure 5:**
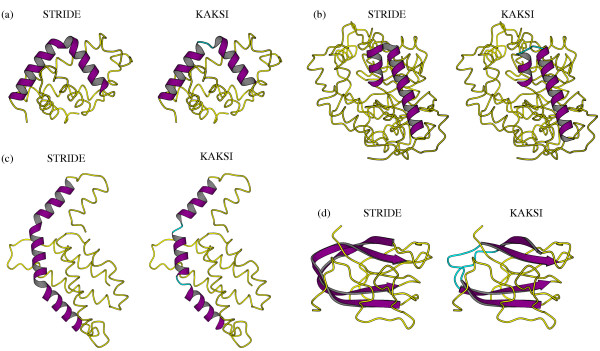
**Examples of disagreement between kaksi and stride**. The divergent assignments are drawn in cartoon representation and highlighted in purple (helix and strand) and cyan (coil assigned by KAKSI). Images are generated with Molscript [46]. Average bending angles (*AverBA*) between local axes computed by HELANAL in long helices are reported, (a): hemoglobin I from the clam Lucina pectinata, PDB code:1b0b, resolution 1.43 Å. STRIDE assignment: *α*-helix from residues 4 to 35, *AverBA *= 15.4°. KAKSI assignment: two helices from 4 to 19, *AverBA *= 3.84° and 21 to 34, *AverBA *= 9.0°. (b): chain A of L(+)-mandelate dehydrogenase from Pseudomonas *putida*, PDB code: 1p4c, resolution 1.35 Å. STRIDE assignment: helix from 308 to 340, *AverBA *= 24.7°. KAKSI assignment: two helices from 308 to 315 and 320 to 341, *AverBA *= 4.3°. (c): chain B of C-phycocyanin from the thermophylic cyanobacterium Synechococcus *elongatus*, PDB code: 1jbo, resolution: 1.45 Å. STRIDE assignment: helix from residues 21 to 62, *AverBA *= 13.1°. KAKSI assignment: 3 helices from 21 to 33, *AverBA *= 4.5°, 35 to 46, *AverBA *= 3.0°, and 48 to 61, *AverBA *= 6.6°. (d): chain A from endo-xylanase from Clostridium *stercorarium*, PDB code: 1od3, resolution: 1 Å. STRIDE assignment: two *β*-strands from 61 to 82 and 116 to 135. KAKSI assignment: four *β*-strands from 61 to 69, 75 to 83, 115 to 122, and 128 to 136.

Figure 2 illustrates the heuristic implemented in KAKSI. We have tested several criteria and combinations of criteria. The final heuristic presented here shows a good agreement with PDB assignments. The principle of the assignment is to test the C*α *distances along the protein to check if they are close to the typical distances in regular secondary structure. The (Φ/Ψ) angles are tested in the same manner. *α*-helix assignment is achieved according to a distance or an angle criterion. The *β*-sheet detection requires the satisfaction of both angle and distance criteria. *α*-helix assignments are corrected whenever kinks are detected. Criteria applied at each step shown on Figure 2 are explained below, in the order they appear in the assignment process. Characteristic values extracted from the *Ref set *are shown in capital. The parameters of the method are : *ε*_*H *_and *ε*_*b *_are used to define thresholds for C*α *distances and *η*_*H *_and *σ*_*b *_are used to define thresholds for the constraints on (Φ/Ψ) angles.

• *Distance criterion for α-helices *(*C1*). All C*α *distances in a sliding window of length *w*_1 _(fixed to 6 in this study) must lie within the interval [*M*_*α *_- *ε *_*H *_× *SD*_*α*_; *M*_*α*_*+ ε*_*H *_× *SD*_*α*_]. *M*_*α *_and *SD*_*α *_represent the mean and standard deviation of C*α *distance distributions in *α*-helices.

• *Angle criterion for α-helices *(*C2*). All (Φ/Ψ) pairs in a sliding window of length *w*_2 _(fixed to 4 in this study) must satisfy the condition (Φ < 0° and -90° < Ψ < 60°) and one pair at least must fall in the highly populated zone of the population matrix, i.e with density> *δ*_*H*_.

• *Kinks in α-helices are detected using two criteria*.

- Kink criterion K1 is based on the values of (Φ/Ψ) dihedral angles. A helix is interrupted at residue *j + *1 if the sum *d*_Φ/Ψ _(*j*, *j + *1) + *d*_Φ/Ψ _(*j *+ 1, *j* + 2) is greater than . *d*_Φ/Ψ _(*j*, *j *+ 1) is analogous to the root mean square deviation on angular value described by Shuchhardt and coll [44]. It measures the distance between dihedral angle pairs of residues *j *and *j + *1 in the Ramachandran map.  is the 95-percentile of the distribution of such distances.

- Kink criterion K2 relies on axes. An axis is fitted along the helix, by minimizing the function  with *n *the number of residues in the helix, *d*_*i *_the distance from the *ith *C*α *to the axis, and *d*_*m *_the mean of the *d*_*i*_*s*. For a perfect (linear) helix the value of *D*_*axis *_is zero and the corresponding vector is the axis of the cylinder circumscribed by backbone atoms. A helix is interrupted if it appears better to fit it with two axes. These two axes must make an angle greater than *θ*_*k *_(*θ*_*k *_fixed to 25° in this study).

• *Distance criterion for β-sheets *(*C3*). All the C*α *distances in two sliding windows of length *w*_3 _(here *w*_3 _= 3) must be in the interval [*M*_*β *_- *ε*_*b *_× *SD*_*β*_; *M*_*β *_+ *ε*_*b *_× *SD*_*β*_]. *M*_*β *_and *SD*_*β *_represent the mean and standard deviation of C*α *distance distributions in *β*-sheets.

• *Angle criterion for β-sheets *(*C4*). For each (Φ/Ψ) angle pair falling in the populated zone of the Ramachandran map (density > 0), we increment a counter *score*(*sheet*) by 1. If a (Φ/Ψ) angle pair of the central residue of a sliding window verifies -120° < Ψ < 50°, then *score*(*sheet*) is reset to zero. The final *score*(*sheet*) must be greater or equal to *σ *_*b*_.

• *Contiguous segments correction, criterion *(*C5*). If a helix and a strand are adjacent, a coil is introduced in between, shortening the helix by one residue.

Empirically, the optimal parameter values are: *ε*_*H *_= 1.96, *η*_*H *_= 2.25, *ε*_*b *_= 2.58 and *σ*_*b *_= 5.

### Comparative methods for secondary structure assignment and reduction to three states

KAKSI assignments are compared to the assignments given by five available methods on the *Comparison sets: *DSSP [21], STRIDE [23], PSEA [27], XTLSSTR [28] and SECSTR [24]. HELIX and SHEET records in PDB files are also considered as an independent assignment method.

When needed, secondary structure assignments are reduced to three classes (H for *α*-helix, b for *β*-strand, c for coil) as follows: DSSP, STRIDE and SECSTR: (H,G,I) = H, (E,b) = b, others (S,T,blank) = c; XTLSSTR: (G,g,H,h) = H, (E,e) = b, others (T,N,P,p,-) = c. PSEA assigns only three states. XTLSSTR possibly provides several alternative assignments for one residue. In that case, only the first assignment is considered. When dealing with NMR structures, only the first model is analyzed.

### Comparison measures

#### Secondary structure content

The secondary structure content of a dataset is measured by the percentage of residues involved in the three structural classes: *α*-helix, *β*-strand and coil.

#### Overall agreement

The *C*_3 _score is the percentage of residues assigned in the same state when comparing two different assignments: *C*_3 _= *N*_*id*_*/N*_*tot *_with *N*_*id *_the number of residues for which both assignments are identical, and *N*_*tot *_the total number of residues with defined secondary structure. It is analogous to the *Q*_3 _score used to evaluate secondary structure prediction.

#### Segment based-agreement

• The mean agreement based on secondary structure segments is measured by the percentage of Segment OVerlap (SOV). We use the SOV definition described by Zemla and coworkers [45]. For state *i *(*α*-helix, *β*-strand or coil) the segment overlap measure is defined as:



with the normalization value *N*(*i*) defined as:



The sums on *S*(*i*) are taken over all the segment pairs in state *i *which overlap by at least one residue. The sum on *S*'(*i*) is taken over the remaining segments in state i found in the reference assignment 1, *len*(*s*_1_) is the number of residues in segment *s*_1_, *minov*(*s*_1_, *s*_2_) is the length of overlap of *s*_1 _and *s*_2_, *maxov*(*s*_1_, *s*_2_) is the total extend for which either of the segments *S*1 and *s*_2 _has a residue in state *i*, and *delta*(*s*_1_, *s*_2_) is defined as:

*min *{*maxov*(*s*_1_, *s*_2_) - *minov*(*s*_1_, *s*_2_); *minov*(*s*_1_, *s*_2_); *int*(*len*(*s*_1_)/2); *int*(*len*(*s*_2_)/2)},

where *min *{*x*1; *x*2; *x*3;...; *xn*} is the minimum of *n *integers. This formula is usually employed to compare a secondary structure prediction (*S*_2_) with a secondary structure description (*S*_1_) taken as reference. The roles of *S*_1 _and *S*_2 _are thus not symmetrical.

• Length of pair of segments used for the SOV computation are collected. A pair is defined each time there is at least one residue in common between assignment X and Y. Unpaired secondary structure elements are ignored in this analysis. These length pairs can be viewed on a bi-plot (length(X) versus length(Y)).

### Helix geometry analysis with an external software

The HELANAL software developed by Kumar and Bansal [33] is dedicated to helix geometry analysis. HELANAL takes as input a PDB file and a description of helix boundaries. It calculates local axes every four residues. The geometry of a helix is determined by the angles between axes and the goodness of fit of the helix trace with a circle or a line. Helices are then classified as kinked (K), linear (L) or curved (C). HELANAL can leave a helix unclassified if its geometry is ambivalent. The minimum length for a helix to be analyzed is nine residues.

In this study, HELANAL is used as an external control of helix geometry. All *α*-helices in the *comparison sets *are submitted to HELANAL analysis. Different assignment methods are used to provide alternate definition of helices boundaries.

## Availability and requirements

• Project name: KAKSI

• Project home page: http://migale.jouy.inra.fr/mig/mig_fr/servlog/kaksi/

• Operating system: Linux

• Programming langage: C

• Other requirements: libxml2 >= 2.6, see ftp://xmlsoft.org/

• License: GNU GPL

• Any restrictions to use by non-academics: no

• Implementation: the software is composed of 2 programs: KAKSI takes a PDB file as input and prints the assigned secondary structure (and other data of intereset) in an XML output K2R reads a KAKSI XML output file and outputs the data in various FASTA format files by default. K2R allows users to easily implement any new output format they whish. a lot of different informations in raw formats (mainly FASTA format).

The source code is available on the project home page.

## List of abbreviations used

3D: three-dimensional, C*α*: backbone *α*-carbon, NMR: Nuclear Magnetic Resonance, PDB: Protein Data Bank.

## Authors' contributions

JM and AM developped the program. GL carried out the comparison between different assignments. JM GL and JFT carried out the analysis. JM, AdB and JFG conceived the study and participated in its design and coordination

## Supplementary Material

Additional File 1 *C*_3 _scores for all datasets. Click here for file

Additional File 2 Graphical views of *C*_3 _scores for the *HRes set*. Click here for file

Additional File 3 SOV scores for all datasets. Click here for file

Additional File 4 Length of pairs of helices and strands on separate plots. Click here for file

Additional File 5 Helix geometry analysis on all datasets. Click here for file

Additional File 6 Urls to retrieve the list of structures used in this study. Click here for file

Additional File 7 Φ/Ψ repartition in helices and strands defined by the PDB. Click here for file

## Figures and Tables

**Table 1:** Distances in *α*-helices. *Core*: distances not involving residues at helix edge. *Termini*: distances involving at least one residue at helix edge. Mean distances, computed on the *HRes set*, are indicated in Å with their standard deviations within parentheses.

Type	Core	Termini
*i *to *i *+ 2	5.49(0.20)	5.54(0.25)
*i *to *i *+ 3	5.30(0.64)	5.36(0.39)
*i *to *i *+ 4	6.33(0.71)	
*i *to *i *+ 5	8.72(0.63)	

**Table 2:** Secondary structure content according to different assignment methods. %*H*: percentage of residues assigned in *α*-helix. %*b*: percentage of residues assigned in *β*-strand. See the text for *β*-strand assignment with *kaksi *using different parameter values on the *LRes *and the *NMR *sets.

Dataset	*HRes set*		*MRes set*		*LRes set*		*NMR set*	
Method	%H	%b	%H	%b	%H	%b	%H	%b
KAKSI	36.8	22.0	38.0	22.5	35.1	19.0	33.5	15.2
PDB	40.5	20.3	41.7	20.9	39.3	18.2	35.5	17.3
DSSP	35.9	22.5	37.3	22.9	35.4	20.4	32.2	17.3
STRIDE	36.4	22.6	38.6	23.3	36.3	21.2	33.7	18.8
PSEA	32.1	23.7	34.2	25.0	33.0	24.4	30.6	22.8
SECSTR	37.2	20.1	38.5	20.4	37.0	18.6	33.3	16.3
XTLSSTR	40.4	19.7	40.9	19.6	35.9	14.4	34.3	14.8

**Table 3:** *C*_3 _scores between different methods on the HRes set

	DSSP	STRIDE	PSEA	SECSTR	XTLSSTR	PDB
KAKSI	82.1%	83.5%	81.5%	81.7%	78.3%	83.4%
DSSP		95.4%	80.1%	93.4%	80.4%	90.8%
STRIDE			81.1%	91.9%	80.8%	89.9%
PSEA				79.8%	75.8%	78.1%
SECSTR					79.6%	87.4%
XTLSSTR						80.7%

**Table 4:** SOV measures between kaksi and other methods on the HRes set. *SOV*_*H*_: SOV for *α*-helix. *SOV*_*b*_: SOV for *β*-strand. KAKSI is taken as reference.

Method	*SOV*_*H*_	*SOV*_*b*_
DSSP	91.7%	92.1%
STRIDE	91.2%	91.9 %
SECSTR	89.0%	83.9%
PSEA	87.5%	82.7%
XTLSSTR	89.3%	73.4%

PDB	88.4%	89.4%

**Table 5:** Helix geometry analyzed by HELANAL on the HRes set. Correction: assignments are corrected by shortening each helix by one residue at each terminus. %L: percentage of helices that are linear according to HELANAL. %C: percentage of helices that are curved according to HELANAL. %K: percentage of helices that are kinked according to HELANAL. N: number of helices submitted to HELANAL.

Method	No correction				With Correction			
Minimum length	11				9 after correction			
	%L	%C	%K	N	%L	%C	%K	N

DSSP	8.3	70.0	21.2	2215	10.9	70.8	17.8	2215
STRIDE	10.1	65.9	23.6	2431	10.8	68.5	20.2	2431
PSEA	10.9	78.5	10.0	2260	11.5	80.0	7.8	2260
SECSTR	8.0	55.7	36.0	2349	10.0	59.7	29.9	2349
XTLSSTR	8.7	58.9	32.1	2618	9.5	61.4	28.9	2618
KAKSI	10.2	66.5	22.8	2442	12.3	72.6	14.5	2442

PDB	11.4	71.1	17.0	2565	11.3	71.5	12.0	2565
