# Supplementary material for: Protein secondary structure assignment revisited: a detailed analysis of different assignment methods
Source: BMC Struct Biol. 2005 Sep 15;5:17. doi: 10.1186/1472-6807-5-17 (PMC1249586; doi:10.1186/1472-6807-5-17)
Supplement: Additional File 1 — C3 scores for all datasets. [file 1472-6807-5-17-S1.pdf]

Table I: C_3 scores between different methods on the *MRes set*

	DSSP	STRIDE	PSEA	SECSTR	XTLSSTR	PDB
KAKSI	82.7%	83.9%	82.0%	82.2%	78.5%	84.0%
DSSP		94.9%	80.8%	93.4%	80.5%	91.5%
STRIDE			81.7%	92.0%	80.7%	90.4%
PSEA				80.5%	76.3%	79.1%
SECSTR					79.8%	88.1%
XTLSSTR						80.7%

Table II: C_3 scores between different methods on the *LRes set*

	DSSP	STRIDE	PSEA	SECSTR	XTLSSTR	PDB
KAKSI	82.6%	83.7%	80.5%	82.5%	77.3%	83.5%
DSSP		93.4%	79.3%	93.1%	77.5%	91.5%
STRIDE			80.1%	91.0%	76.9%	89.3%
PSEA				78.8%	73.4%	77.9%
SECSTR					76.6%	88.3%
XTLSSTR						77.3%

Table III: C_3 scores between different methods on the *NMR set*

	DSSP	STRIDE	PSEA	SECSTR	XTLSSTR	PDB
KAKSI	84.7%	85.5%	80.1%	84.9%	78.7%	85.2%
DSSP		94.3%	81.0%	94.4%	79.9%	92.0%
STRIDE			81.6%	92.3%	79.4%	90.3%
PSEA				80.7%	74.5%	79.1%
SECSTR					78.9%	89.3%
XTLSSTR						78.5%
