# Supplementary material for: Protein secondary structure assignment revisited: a detailed analysis of different assignment methods
Source: BMC Struct Biol. 2005 Sep 15;5:17. doi: 10.1186/1472-6807-5-17 (PMC1249586; doi:10.1186/1472-6807-5-17)
Supplement: Additional File 2 — Graphical views of C3 scores for the HRes set. [file 1472-6807-5-17-S2.pdf]

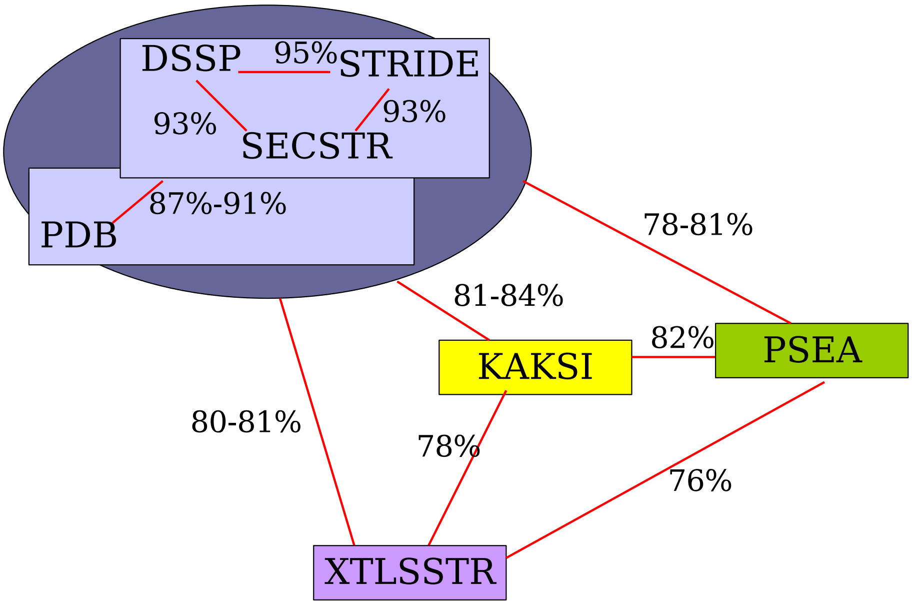

Figure 1: Graphical view of the C_3 scores in the *HRes set*

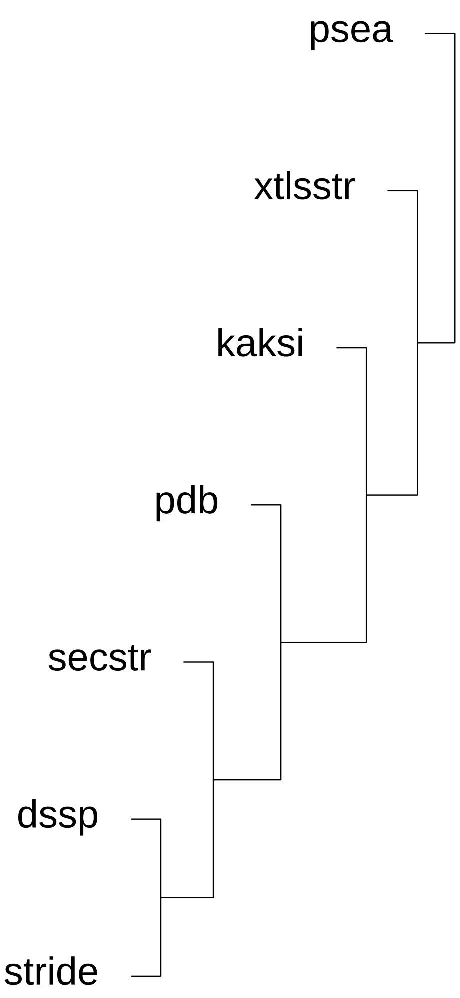

Figure 2: Tree obtained with a hierarchical clustering with distances equal to 100-C_3 in the *HRes set*
