# Supplementary material for: Protein secondary structure assignment revisited: a detailed analysis of different assignment methods
Source: BMC Struct Biol. 2005 Sep 15;5:17. doi: 10.1186/1472-6807-5-17 (PMC1249586; doi:10.1186/1472-6807-5-17)
Supplement: Additional File 3 — SOV scores for all datasets. [file 1472-6807-5-17-S3.pdf]

Table IV: SOV measures between KAKSI and other methods on various datasets with KAKSI as reference

Data set	*MRes set*		*LRes set*		*NMR set*	
Method	SOV_H [a]	SOV_b [b]	SOV_H	SOV_b	SOV_H	SOV_b
DSSP	91.4%	92.4%	90.6%	88.6%	91.6%	89.1%
STRIDE	89.9%	91.4%	89.9%	90.0%	92.1%	91.2%
SECSTR	87.5%	83.2%	88.4%	84.2%	90.8%	85.0%
PSEA	87.9%	84.3%	88.3%	82.6%	89.6%	82.3%
XTLSSTR	87.3%	73.2%	86.2%	63.5%	88.9%	63.5%
PDB	89.1%	89.8%	89.3%	86.4%	91.0%	91.1%

[a] SOV for α-helix
[b] SOV for β-strand
