# Supplementary material for: Protein secondary structure assignment revisited: a detailed analysis of different assignment methods
Source: BMC Struct Biol. 2005 Sep 15;5:17. doi: 10.1186/1472-6807-5-17 (PMC1249586; doi:10.1186/1472-6807-5-17)
Supplement: Additional File 4 — Length of pairs of helices and strands on separate plots. [file 1472-6807-5-17-S4.pdf]

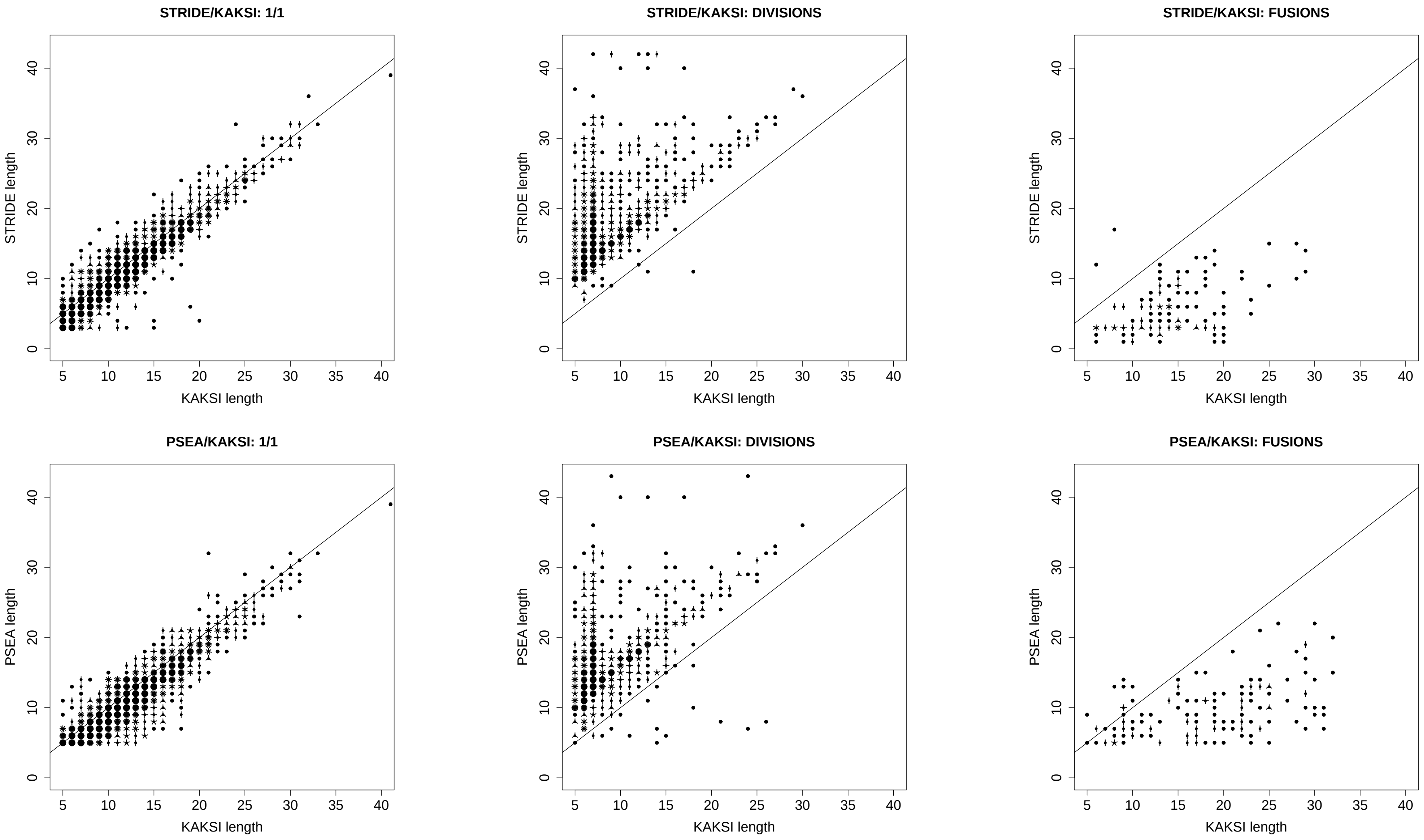

Figure 3: Length for pair of **helices** when comparing STRIDE and KAKSI assignments, and PSEA and KAKSI assignments. The 3 cases (one-to-one, fusion or division events) are plotted on separate graphs.

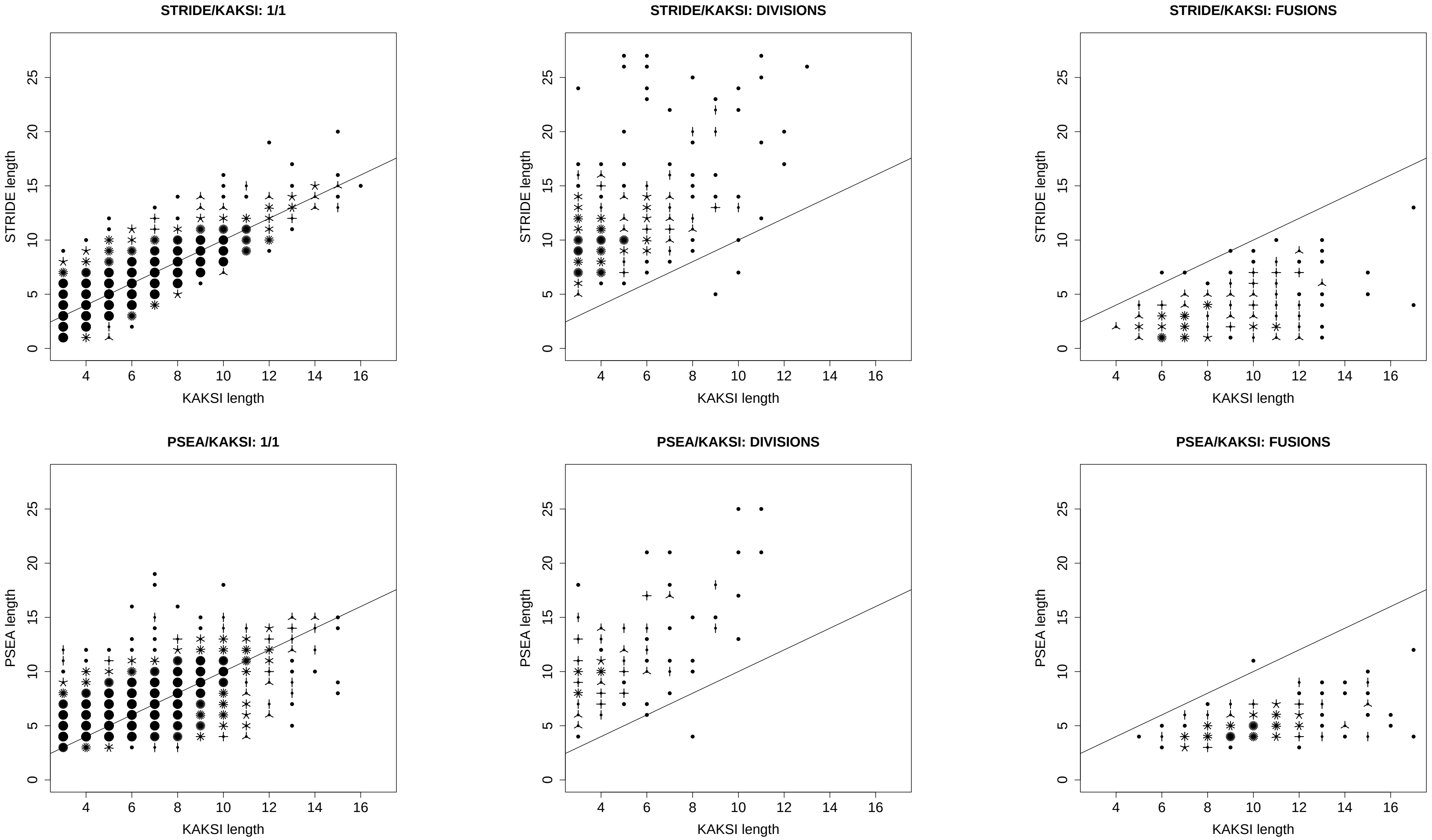

Figure 4: Length for pair of **strands** when comparing STRIDE and KAKSI assignments, and PSEA and KAKSI assignments. The 3 cases (one-to-one, fusion or division events) are plotted on separate graphs.
