# Supplementary material for: Protein secondary structure assignment revisited: a detailed analysis of different assignment methods
Source: BMC Struct Biol. 2005 Sep 15;5:17. doi: 10.1186/1472-6807-5-17 (PMC1249586; doi:10.1186/1472-6807-5-17)
Supplement: Additional File 5 — Helix geometry analysis on all datasets. [file 1472-6807-5-17-S5.pdf]

Table V: Helix geometry analyzed by HELANAL on the *MRes set*

Method	No correction				With Correction [a]			
Minimum length	11				9 after correction			
	%L[b]	%C[c]	%K[d]	N[e]	%L	%C	%K	N
DSSP	10.79	64.43	24.15	2381	12.47	66.74	20.20	2381
STRIDE	10.99	64.04	24.43	2567	12.66	65.52	21.08	2567
PSEA	12.18	73.22	13.81	2390	13.26	74.31	11.59	2390
SECSTR	9.10	54.93	35.57	2494	11.15	58.38	29.99	2494
XTLSSTR	9.81	60.30	29.52	2690	11.71	61.12	26.88	2690
KAKSI	11.26	64.02	23.95	2593	13.23	70.15	15.77	2593
PDB	12.23	65.98	21.03	2763	13.86	69.56	15.92	2763

[a]assignments are corrected by shortening each helix by one residue at each extremity
[b]percentage of helices that are linear according to HELANAL
[c]percentage of helices that are curved according to HELANAL
[d]percentage of helices that are kinked according to HELANAL
[e]number of helices submitted to HELANAL

Table VI: Helix geometry analyzed by HELANAL on the *LRes set*

Method	No correction				With Correction			
Minimum length	11				9 after correction			
	%L	%C	%K	N	%L	%C	%K	N
DSSP	15.04	54.60	29.57	1390	18.55	57.80	23.08	1391
STRIDE	14.06	50.65	34.47	1465	16.38	54.81	27.99	1465
PSEA	15.55	62.41	20.90	1402	19.40	63.41	15.98	1402
SECSTR	12.07	44.10	42.74	1467	15.80	48.84	34.67	1468
XTLSSTR	13.57	53.19	31.64	1378	17.34	53.41	27.65	1378
KAKSI	16.17	57.20	25.71	1416	20.20	61.09	17.58	1416
PDB	14.58	54.98	29.74	1577	17.44	59.80	21.81	1577

Table VII: Helix geometry analyzed by HELANAL on the *NMR set*

Method	No correction				With Correction			
Minimum length	11				9 after correction			
	%L	%C	%K	N	%L	%C	%K	N
DSSP	7.20	46.54	45.43	361	11.63	52.35	35.18	361
STRIDE	7.07	40.91	51.26	396	11.87	45.20	41.92	396
PSEA	7.99	51.24	39.94	363	13.22	53.99	31.40	363
SECSTR	6.53	37.86	54.83	383	8.36	46.48	44.39	383
XTLSSTR	6.15	47.33	45.72	374	7.75	54.81	35.56	374
KAKSI	7.32	43.94	47.98	396	13.38	50.00	35.86	396
PDB	9.15	39.44	50.70	426	11.74	49.30	38.50	426
