# Supplementary material for: Protein secondary structure assignment revisited: a detailed analysis of different assignment methods
Source: BMC Struct Biol. 2005 Sep 15;5:17. doi: 10.1186/1472-6807-5-17 (PMC1249586; doi:10.1186/1472-6807-5-17)
Supplement: Additional File 6 — Urls to retrieve the list of structures used in this study. [file 1472-6807-5-17-S6.pdf]

The list of PDB files used in our study are available at url `http://genome.jouy.inra.fr/~jumartin/kaksi/sup_data/`

- *Ref set*: `Ref_list.txt`,
- *HRes set*: `HRes_list.txt`,
- *MRes set*: `MRes_list.txt`,
- *LRes set*: `LRes_list.txt`,
- *NMR set*: `NMR_list.txt`.

No that these are the final lists, i.e., PDB files that are successfully processed by all assignment programms and correctly post-processed. PDB files lacking the HELIX and SHEET fields are removed.
