# Supplementary material for: Protein secondary structure assignment revisited: a detailed analysis of different assignment methods
Source: BMC Struct Biol. 2005 Sep 15;5:17. doi: 10.1186/1472-6807-5-17 (PMC1249586; doi:10.1186/1472-6807-5-17)
Supplement: Additional File 7 — Φ/Ψ repartition in helices and strands defined by the PDB. [file 1472-6807-5-17-S7.pdf]

Angles in α-helices

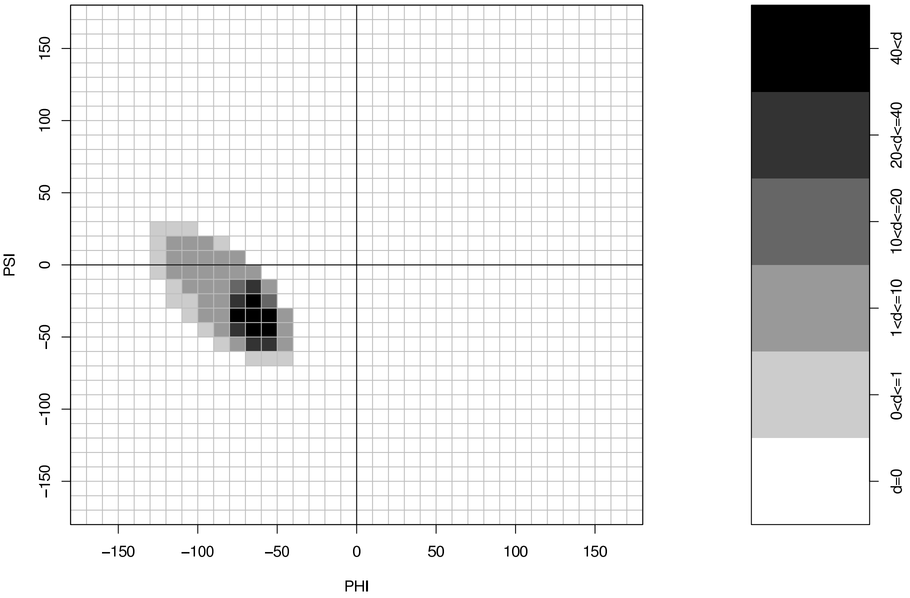

Figure 5: (Φ/Ψ) angles population map of α-helices

Angles in β-strands

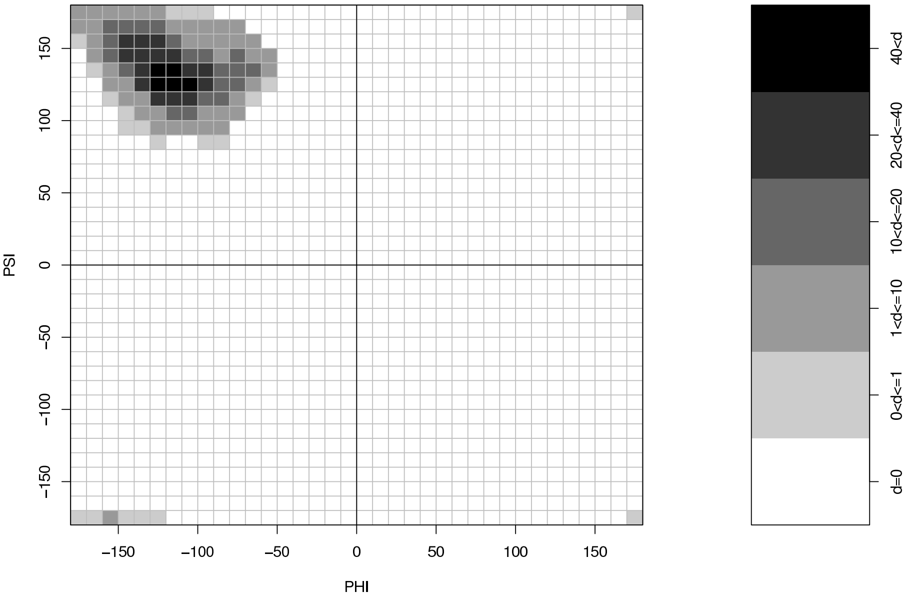

Figure 6: (Φ/Ψ) angles population map in β-strands
